# Postoperative Outcomes of the Desarda Technique Versus Lichtenstein Mesh Repair for Inguinal Hernias: A Systematic Review and Meta-Analysis

**DOI:** 10.7759/cureus.91388

**Published:** 2025-09-01

**Authors:** Jahanzaib Saeed, Zohaib Jamal, Asher Siddiqui, Muhammad Muawaz, Talha Saeed, Rajesh K Jain

**Affiliations:** 1 Department of General Surgery, The Shrewsbury and Telford Hospital NHS Trust, Shrewsbury, GBR; 2 Department of Surgery, Wrightington, Wigan and Leigh NHS Foundation Trust, Wigan, GBR; 3 Department of Surgery, Allied Hospital, Faisalabad, PAK; 4 Department of Surgery, Federal Medical College, Islamabad, PAK; 5 Department of Upper GI and Bariatric Surgery, The Shrewsbury and Telford Hospital NHS Trust, Shrewsbury, GBR

**Keywords:** desarda repair, desarda technique, inguinal hernia repair, lichenstein repair, lichtenstein mesh repair, lichtenstein tension-free mesh repair, mesh

## Abstract

Inguinal hernias represent a prevalent surgical condition worldwide and constitute a significant proportion of elective general surgical procedures. While the Lichtenstein mesh repair has become the standard technique due to its tension-free approach, it is associated with several mesh-related complications, including chronic postoperative pain, seroma, scrotal edema, and foreign body sensation. To overcome these limitations, the Desarda technique was developed as a tissue-based, mesh-free alternative that reinforces the posterior wall of the inguinal canal using a strip of the external oblique aponeurosis. This systematic review compares postoperative complications between the Lichtenstein mesh repair and Desarda techniques to inform best practices in inguinal hernia repair.

This systematic review was conducted according to the Cochrane Handbook for Systematic Reviews of Interventions and reported in line with the Preferred Reporting Items for Systematic Reviews and Meta-Analyses (PRISMA) guidelines. A comprehensive search was performed across PubMed, Cochrane Library, Excerpta Medica database (EMBASE), Emcare, Medical Literature Analysis and Retrieval System Online (MEDLINE), and Ovid, without language or date restrictions. Randomized controlled trials (RCTs) comparing Desarda and Lichtenstein mesh repair for primary inguinal hernia in adults were included. Data on postoperative complications were independently extracted by two reviewers. Risk of bias was assessed using the Risk of Bias 2 (ROB 2) tool (The Cochrane Collaboration, London, United Kingdom). Statistical analysis was performed using RevMan 5.4 (The Cochrane Collaboration, 2020), with risk ratios (RR) and 95% confidence intervals (CI) calculated for binary outcomes. Heterogeneity was assessed using the chi-square and I² statistics.

A total of 23 RCTs were included, comprising 2,425 patients, 1,201 of whom underwent the Desarda repair and 1,233 who underwent Lichtenstein mesh repair. The Desarda technique was associated with significantly lower rates of scrotal edema (RR = 0.52, 95% CI: 0.34-0.78, p = 0.002), seroma formation (RR = 0.68, 95% CI: 0.47-0.99, p = 0.04), foreign body sensation (RR = 0.61, 95% CI: 0.42-0.88, p = 0.009), and chronic postoperative pain (RR = 0.26, 95% CI: 0.15-0.45, p < 0.00001). While the Desarda group also showed lower rates of recurrence, wound infection, wound hematoma, and loss of sensation, these differences were not statistically significant and should be interpreted with caution.

The findings suggest that the Desarda technique may be preferable to the Lichtenstein mesh repair for primary inguinal hernia, as it is associated with significantly lower rates of chronic postoperative pain, seroma, scrotal edema, and foreign body sensation. Although other complications, such as loss of sensation, recurrence, wound infection, and hematoma, were also less frequent with the Desarda technique, these differences were not statistically significant. Overall, the outcomes were largely comparable.

## Introduction and background

Inguinal hernias represent one of the most frequently encountered conditions in surgical practice globally, with more than 20 million individuals undergoing operative repair each year [1]. The Bassini technique, introduced in the 1880s, was the first method employed for hernia repair [2]. Over subsequent decades, various surgical approaches were developed, including the Modified Bassini, Shouldice, and Darning techniques [2]. The primary objective was to achieve a tension-free hernia repair while minimizing postoperative complications [2]. In recent decades, more advanced techniques have been introduced, including laparoscopic approaches and the conventional Lichtenstein mesh repair [3]. While this strategy has lowered recurrence rates, mesh implantation is associated with specific complications. For example, chronic postoperative groin pain has been reported in up to 30% of patients following mesh repair, and foreign body sensation is experienced by approximately 10% to 15% [4]. Other complications include seroma formation, scrotal edema, and wound infection, all of which may affect quality of life.

To address the complications associated with mesh-based hernia repairs, a novel tissue-based technique was introduced in the early 21st century by Dr. Mohan Desarda [5]. This method involves the use of a strip of the external oblique aponeurosis to reinforce the posterior wall of the inguinal canal, thereby eliminating the need for synthetic mesh. International guidelines acknowledge tissue-based techniques such as Desarda as reasonable alternatives in specific clinical scenarios, particularly in resource-limited settings or where synthetic mesh is contraindicated [6]. By utilizing the patient’s native tissue, the Desarda technique aims to reduce the incidence of mesh-related complications, such as chronic postoperative pain, infection, and foreign body sensation. Furthermore, it is relatively simple to learn, requires minimal additional dissection, and may be particularly advantageous in resource-limited settings where mesh availability or affordability is an issue [5].

This systematic review aims to compare postoperative complications between the mesh-based Lichtenstein mesh repair and the tissue-based Desarda technique for inguinal hernia repair, focusing on outcomes such as pain, infection, seroma, scrotal edema, and recurrence to guide clinical practice. Although several systematic reviews have examined mesh versus non-mesh repairs in general, robust meta-analyses directly comparing the Desarda technique with the Lichtenstein mesh repair technique remain limited. Furthermore, current international guidelines continue to recommend mesh repair as the standard, despite concerns regarding mesh-related complications and cost-effectiveness in low-resource settings. These gaps underscore the need for the present systematic review and meta-analysis.

## Review

Methodology

This review was conducted following the methodological guidance of the Cochrane Handbook for Systematic Reviews of Interventions. Reporting was performed in accordance with the Preferred Reporting Items for Systematic Reviews and Meta-Analyses (PRISMA) guidelines [7].

Search Strategy

A comprehensive literature search was conducted independently by two reviewers across the following databases: PubMed, Cochrane Library, Excerpta Medica database (EMBASE), Emcare, Medical Literature Analysis and Retrieval System Online (MEDLINE), and Ovid. The final search was performed on 31^st^ March, 2025. No language or publication date restrictions were applied to ensure a broad and inclusive selection of relevant studies. The search strategy included the following terms and Boolean operators: “inguinal hernia” OR “groin hernia” AND “Desarda” OR “mesh” OR “Lichtenstein” AND “postoperative complications” OR “complications.”

Eligibility Criteria

Studies were included based on the following criteria: (1) participants aged over 18 years; (2) patients with primary inguinal hernia; (3) randomized controlled trials (RCTs); and (4) studies directly comparing the Desarda and Lichtenstein mesh repair techniques.

Exclusion criteria were (1) participants younger than 18 years; (2) studies involving recurrent or strangulated hernias; (3) case reports, cohort studies, and evaluation studies; and (4) studies employing laparoscopic techniques.

Data Extraction

Data were independently extracted by two reviewers using a predefined extraction form. The following variables were collected from the included studies: study design, sample size, country, year of publication, gender distribution, and the number of participants undergoing Desarda or Lichtenstein mesh repair. Postoperative outcomes assessed included duration of hospital stay, chronic postoperative pain, wound infection, wound hematoma, loss of sensation, foreign body sensation, seroma formation, recurrence, and scrotal edema. 

Risk of Bias Assessment

The risk of bias for the included RCTs was evaluated using the Risk of Bias 2 (ROB 2) tool (2019 version, The Cochrane Collaboration, London, United Kingdom) [8]. The following domains were assessed: (1) bias arising from the randomization process; (2) deviations from the intended interventions; (3) missing outcome data; (4) measurement of outcomes; and (5) selection of the reported results. Overall, the studies were judged to have a low risk of bias. Final assessments were made by consensus between two independent reviewers.

Statistical Analysis

Risk ratios (RR) with 95% confidence intervals (CIs) were calculated for all binary outcomes. A p-value of <0.05 was considered statistically significant. Heterogeneity among studies was assessed using the chi-square test and quantified using the I² statistic. An I² value close to 0 indicated low variability among studies. Sensitivity and publication bias analyses were considered but not feasible due to the limited number of studies available for most outcomes. All statistical analyses were conducted using RevMan version 5.4 (The Cochrane Collaboration, 2020) [9].

Results

Study Selection 

A total of 4,496 records were identified through database searches. Following the removal of duplicates, title and abstract screening, and full-text review, 76 studies were assessed for eligibility. Of these, 23 studies met the inclusion criteria and were included in the systematic review.

**Figure 1:**
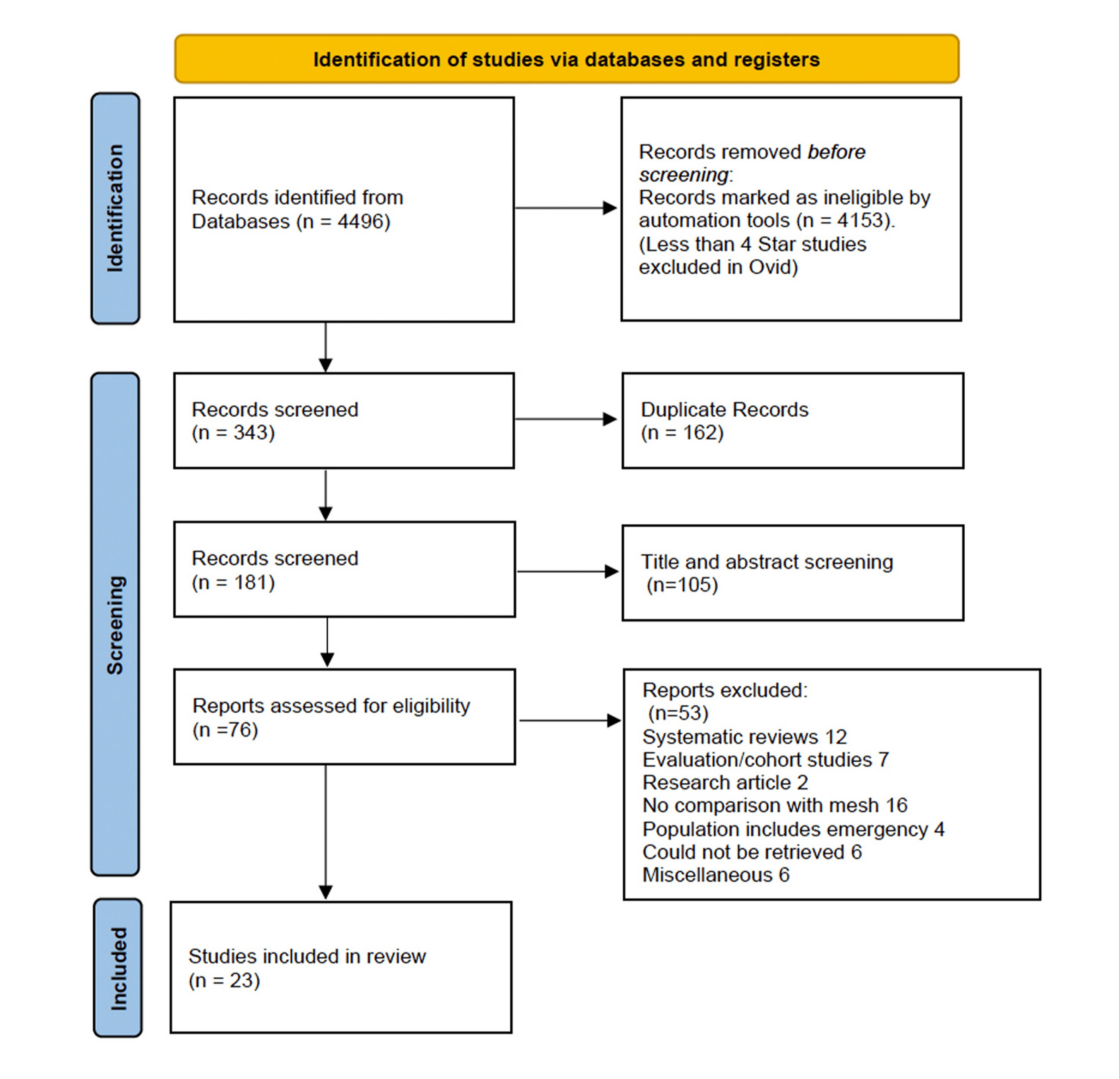
A PRISMA flow diagram outlining the study selection process PRISMA: Preferred Reporting Items for Systematic Reviews and Meta-Analyses

*Study Characteristics* The final analysis comprised 2,425 patients, with 1,201 undergoing Desarda repair and 1,233 undergoing Lichtenstein mesh repair. All included patients had primary inguinal hernias. Although some studies reported cases of bilateral hernias, it was clearly stated that only one side was operated on in each case. The study selection process is illustrated in the PRISMA flow diagram Figure 1 and study characteristics are summarized in Table 1.

**Table 1:** Study characteristics RCT: randomized controlled trial; CTRI: International Clinical Trials Registry

Study ID	Study design/Method of randomisation	Country	Year	Size of the population	Desarda technique	Lichtenstein mesh repair technique
Afzal et al. [10]	RCT/Computer-generated sequence	Pakistan	2017	70	35	35
Ahmad et al. [11]	RCT/Block randomization	Pakistan	2022	250	125	125
Ahmed et al. [12]	RCT/Not specified	Pakistan	2020	100	50	50
Arafa et al. [13]	RCT/Computer-generated sequence	Egypt	2019	80	40	40
Bhatti et al. [14]	RCT/Not specified	Pakistan	2015	200	100	100
CTRI 008373 [15]	RCT/Computer-generated sequence	India	2023	56	26	30
CTRI 015783 [16]	RCT/Computer-generated sequence	India	2019	123	62	61
Dhananjaya et al. [17]	RCT/Not specified	United Kingdom	2022	72	36	36
Gaur et al. [18]	RCT/Computer-generated sequence (web-based)	India	2022	96	46	50
Jain et al. [19]	RCT/Computer-generated sequence	England	2021	84	44	40
Kamran et al. [20]	RCT/Lottery method	Pakistan	2021	60	30	30
Manyilirah et al. [21]	RCT/Double-blind; randomization method not specified	Uganda	2012	101	50	51
Maurya et al. [22]	RCT/Not specified	India	2024	100	50	50
Mehmood et al. [23]	RCT/Block randomization	Pakistan	2022	64	32	32
Mirza et al. [24]	RCT/Computer-generated sequence (web-based)	Pakistan	2023	60	30	30
Muhammad et al. [25]	RCT/Lottery method	Pakistan	2019	60	30	30
Paliwal et al. [26]	RCT/Not specified	India	2022	100	50	50
Paul et al. [27]	RCT/Not specified	India	2022	132	62	76
Pradhan et al. [28]	RCT(comparative)/Not specified	India	2023	71	35	36
Saraswat et al. [29]	RCT/Lottery method (envelope technique)	India	2023	60	30	30
Szopinski et al. [30]	RCT/Computer generation sequence	Poland	2012	205	105	103
Verma et al. [31]	RCT/Random allocation; method not specified	India	2023	138	62	76
Youssef et al. [32]	RCT/Not specified	United Kingdom	2015	143	71	72

Risk of Bias Summary

**Figure 2:**
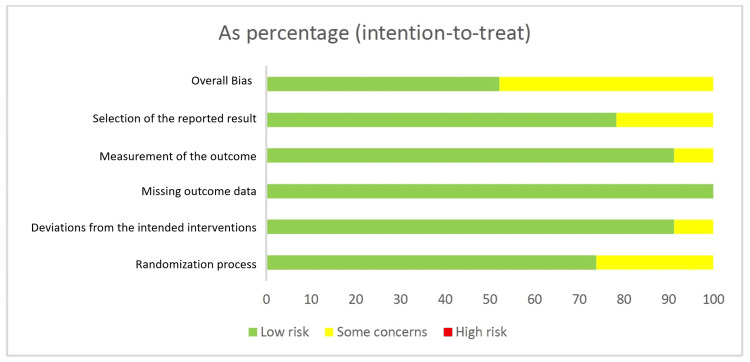
Domain-based risk of bias summary across included RCTs (intention-to-treat analysis) RCTs: randomized controlled trials

**Figure 3:**
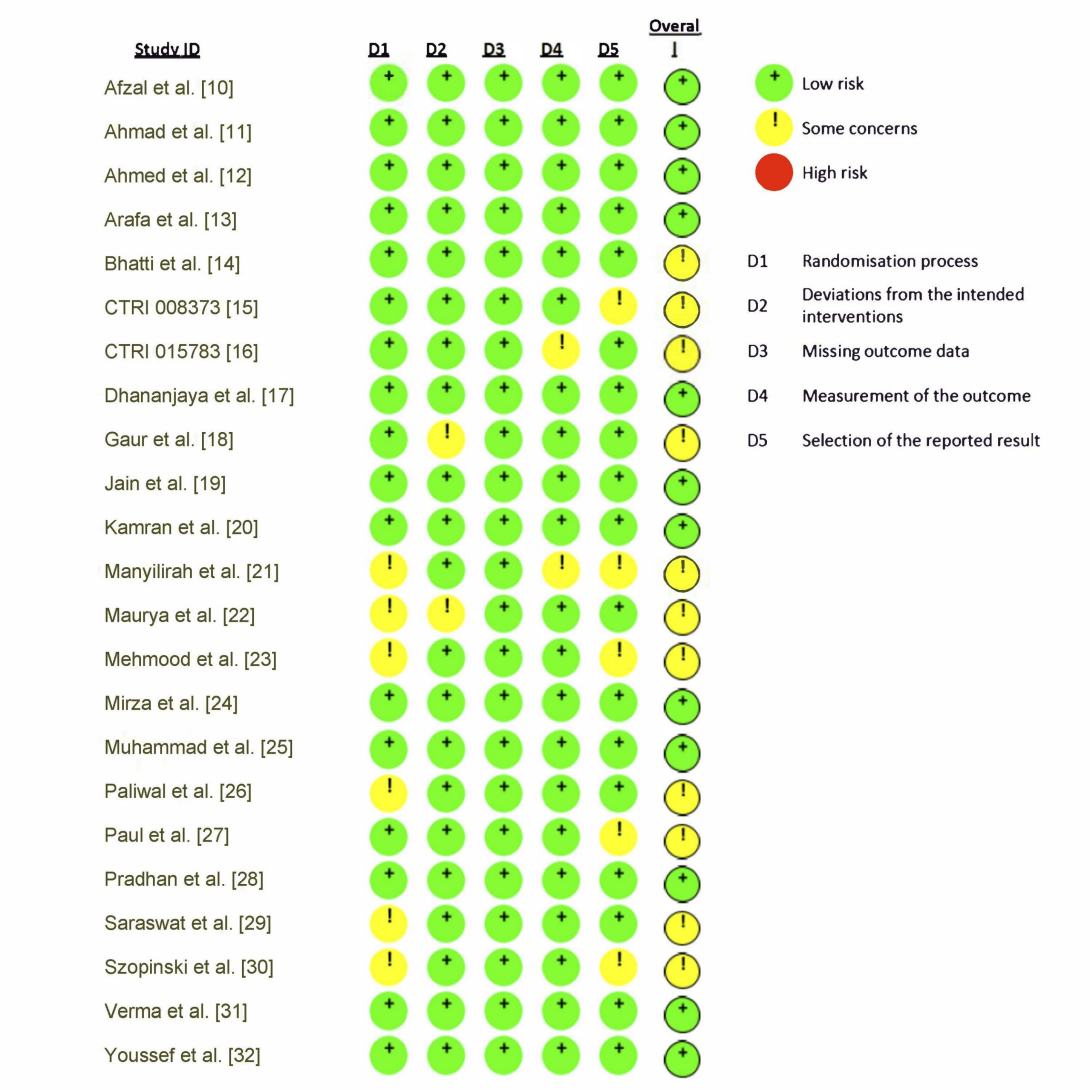
Risk of bias assessment (Risk of Bias 2 (ROB 2)) across the 23 included studies using five domains (D1–D5). CTRI: International Clinical Trials Registry

The individual quality appraisal of each study is presented in Figures 2, 3. Although all included studies were RCTs, the quality of reporting was variable. While several trials clearly described randomization methods (e.g., computer-generated sequences, block randomization, or lottery methods), others simply stated that randomization was performed without specifying the process. Similarly, blinding was infrequently reported, with only a few trials providing details of single- or double-blind designs. This variability accounts for the classification of 11 studies as having “some concerns” in the ROB 2 assessment.

Primary Outcomes

**Figure 4:**
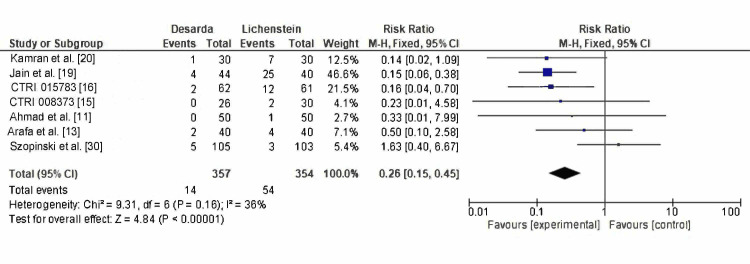
Comparison of chronic postoperative pain rate between the Desarda technique and Lichtenstein mesh repair groups CTRI: International Clinical Trials Registry

Chronic postoperative pain: Chronic postoperative pain was reported in seven RCTs, occurring in 14 of 357 patients in the Desarda group and 54 of 354 patients in the Lichtenstein mesh repair group. The pooled RR was 0.26 (95% CI: 0.15-0.45; p < 0.00001), with a heterogeneity level of I² = 36%, as shown in Figure 4. These results are statistically significant and suggest that the Desarda technique is associated with a substantially lower risk of chronic postoperative pain compared to the Lichtenstein mesh repair technique.

**Figure 5:**
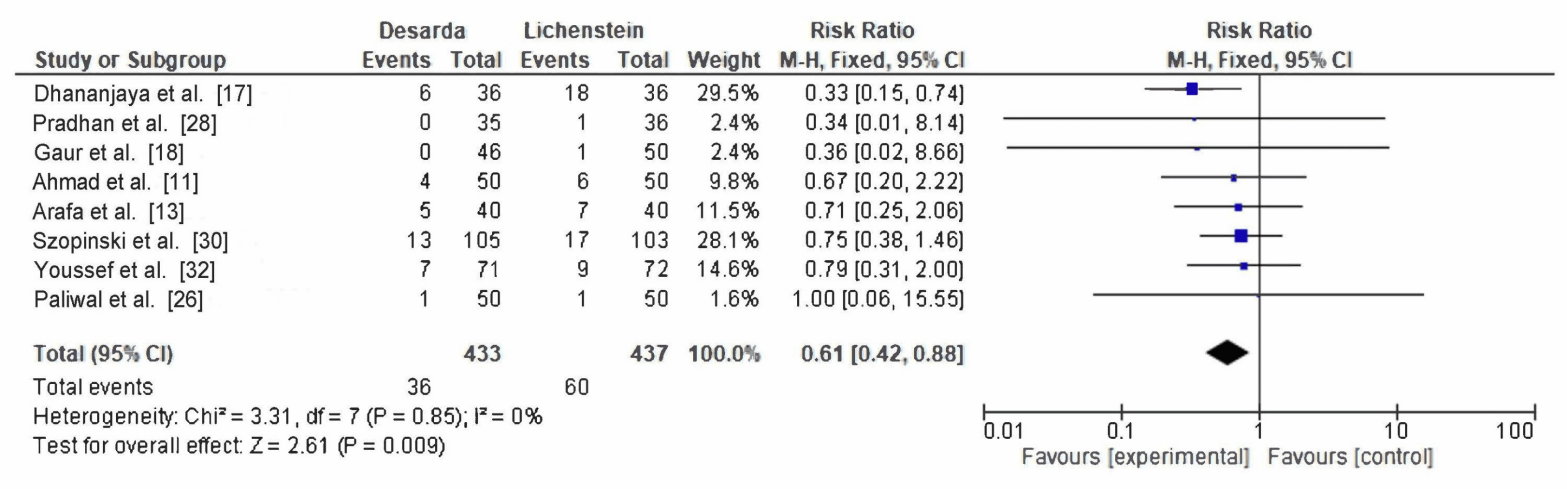
Comparison of foreign body sensation reported between the Desarda technique and Lichtenstein mesh repair groups

Foreign body sensation: Foreign body sensation was evaluated in eight RCTs, reported in 36 of 433 patients who were in the Desarda group and 60 of 437 patients in the Lichtenstein mesh repair group. The pooled RR was 0.61 (95% CI: 0.42-0.88; p = 0.009), with no observed heterogeneity (I² = 0%), as shown in Figure 5. The findings are statistically significant and indicate that the Desarda technique is associated with a reduced incidence of foreign body sensation.

**Figure 6:**
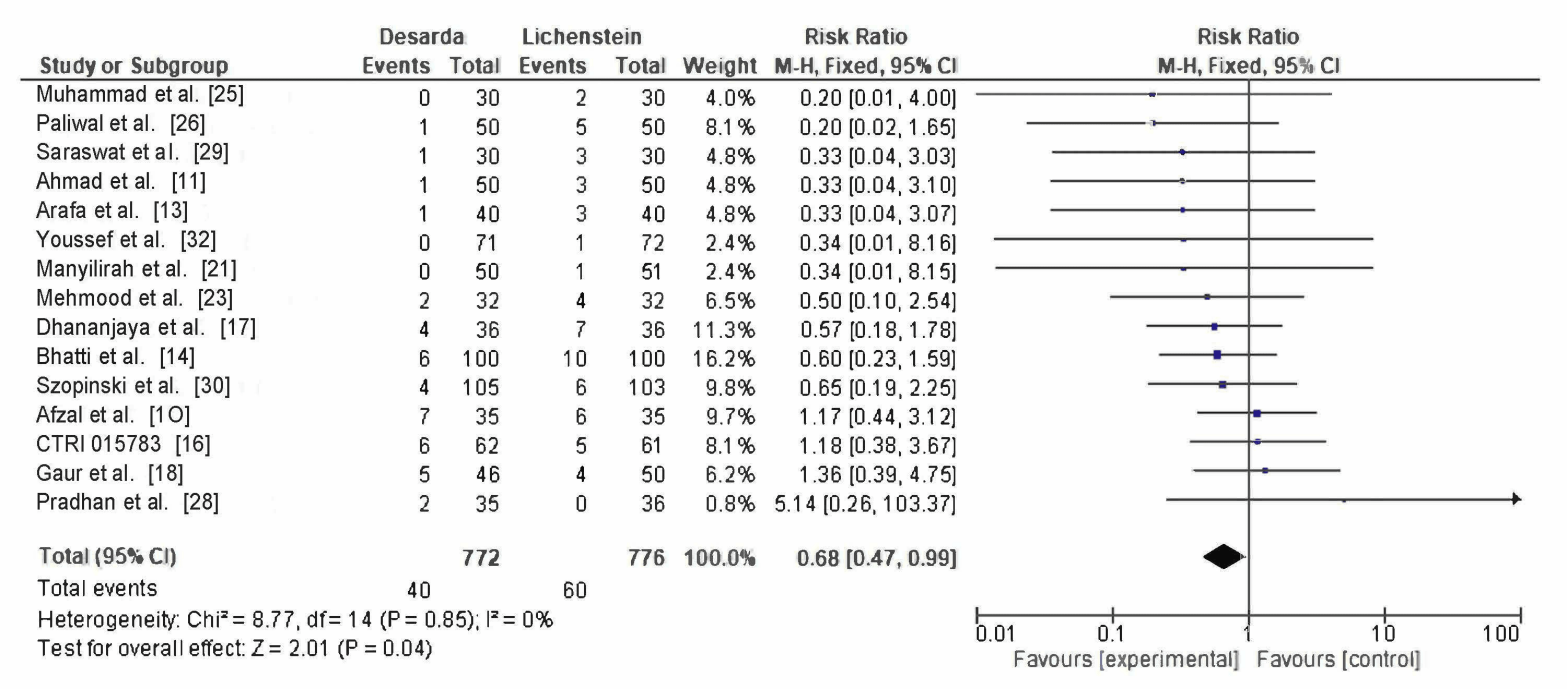
Comparison of the incidence of seroma formation between between the Desarda technique and Lichtenstein mesh repair groups CTRI: International Clinical Trials Registry

Seroma formation: Fifteen RCTs reported on seroma formation, with 40 of 772 patients in the Desarda group and 60 of 776 patients in the Lichtenstein mesh repair group. The calculated RR was 0.68 (95% CI: 0.47-0.99; p = 0.04), with I² = 0%, as shown in Figure 6. The results are statistically significant, suggesting that the Desarda technique is associated with a lower risk of seroma formation.

**Figure 7:**
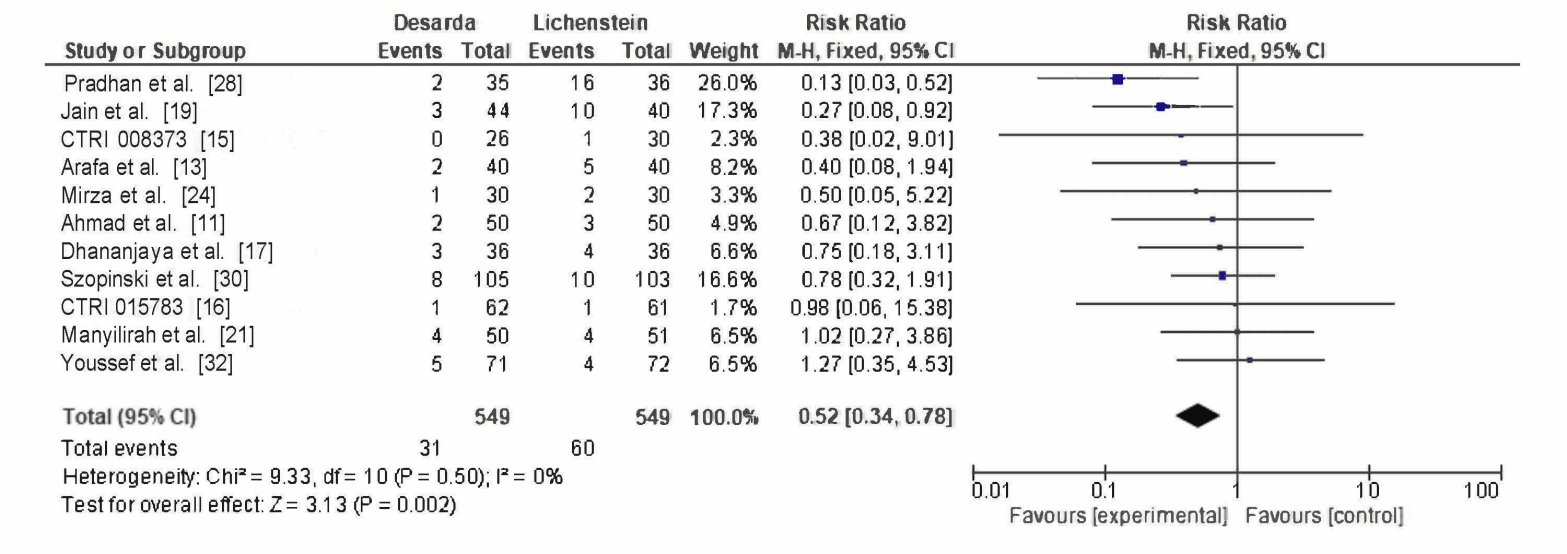
Comparison of scrotal edema occurence between between the Desarda technique and Lichtenstein mesh repair groups CTRI: International Clinical Trials Registry

Scrotal edema: Scrotal edema was documented in 11 RCTs, affecting 31 of 549 patients in the Desarda group and 60 of 549 patients in the Lichtenstein mesh repair group. The pooled RR was 0.52 (95% CI: 0.34-0.78; p = 0.002), with no heterogeneity (I² = 0%), as shown in Figure 7. These findings are statistically significant and indicate that the Desarda technique significantly reduces the risk of scrotal edema compared to the Lichtenstein mesh repair technique.

**Figure 8:**
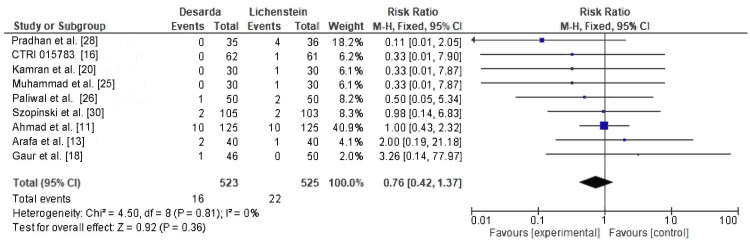
Comparison of recurrence rates between the Desarda technique and Lichtenstein mesh repair groups CTRI: International Clinical Trials Registry

Recurrence:** **Recurrence rates were reported in nine RCTs, occurring in 16 of 523 patients in the Desarda group and 22 of 525 patients in the Lichtenstein mesh repair group. The RR was 0.76 (95% CI: 0.42-1.37; p = 0.36), with I² = 0%, as shown in Figure 8. These results are not statistically significant, and the wide CI, coupled with small sample sizes in some studies, limits the reliability of the findings. However, there is a slight trend favoring the Desarda technique.

**Figure 9:**
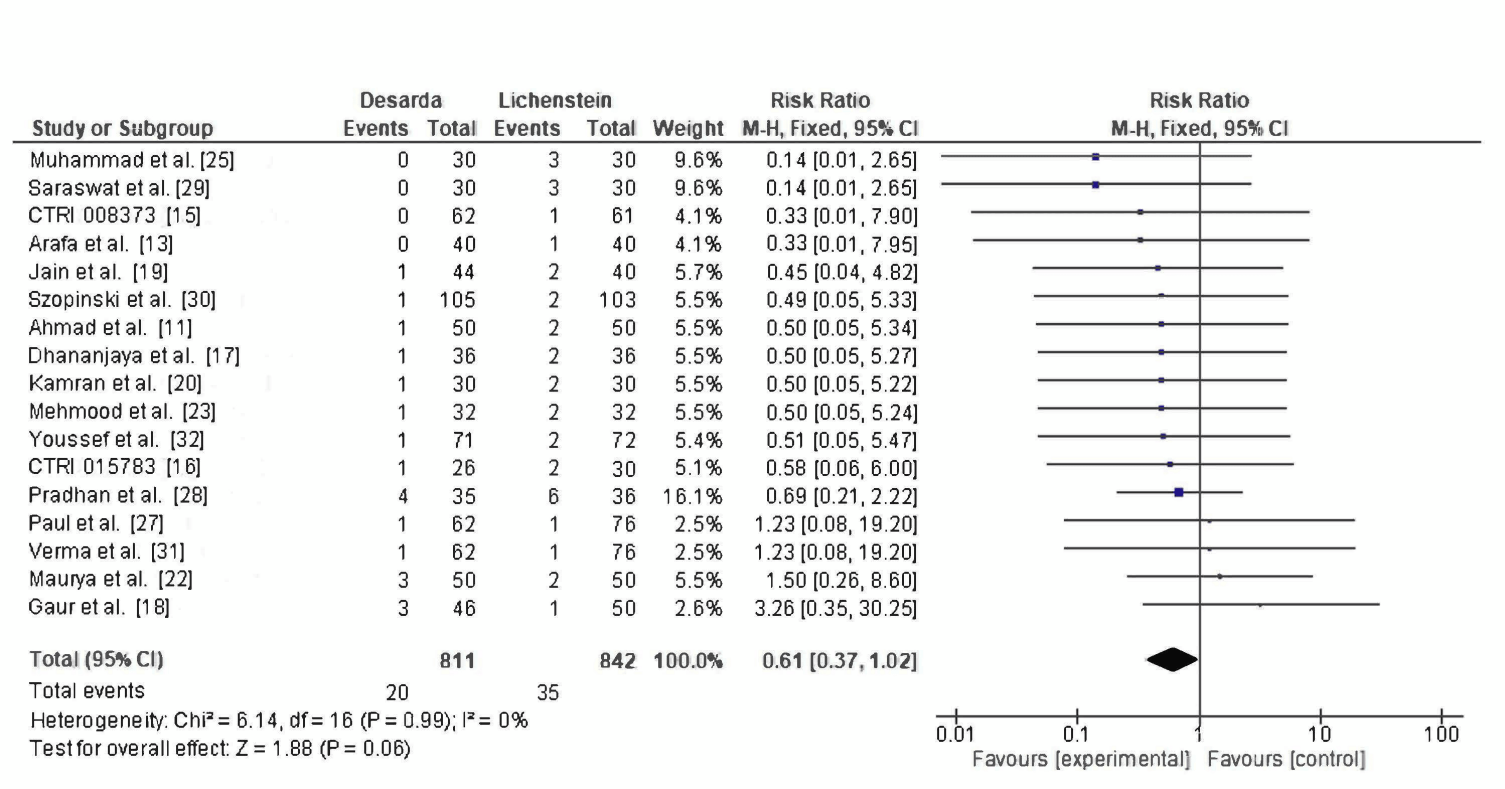
Comparison of wound infection incidence between the Desarda technique and Lichtenstein mesh repair groups CTRI: International Clinical Trials Registry

Wound infection: Wound infection was assessed in 17 RCTs, reported in 20 of 811 patients in the Desarda group and 35 of 842 patients in the Lichtenstein mesh repair group. The RR was 0.61 (95% CI: 0.37-1.02; p = 0.06), with I² = 0%, as shown in Figure 9. While the trend favors the Desarda technique, the result does not reach statistical significance, and no definitive conclusion can be drawn.

**Figure 10:**
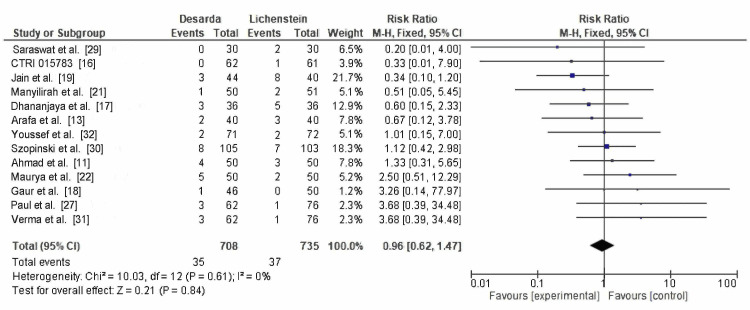
Comparison of wound hematoma occurence between between the Desarda technique and Lichtenstein mesh repair groups CTRI: International Clinical Trials Registry

Wound hematoma: Thirteen RCTs reported wound hematoma, with 35 of 708 patients in the Desarda group and 37 of 735 patients in the Lichtenstein mesh repair group. The RR was 0.96 (95% CI: 0.62-1.47; p = 0.84), with I² = 0%, as shown in Figure 10. Although the results are statistically reliable, they are not clinically meaningful, indicating no significant difference in wound hematoma formation between the two techniques.

**Figure 11:**
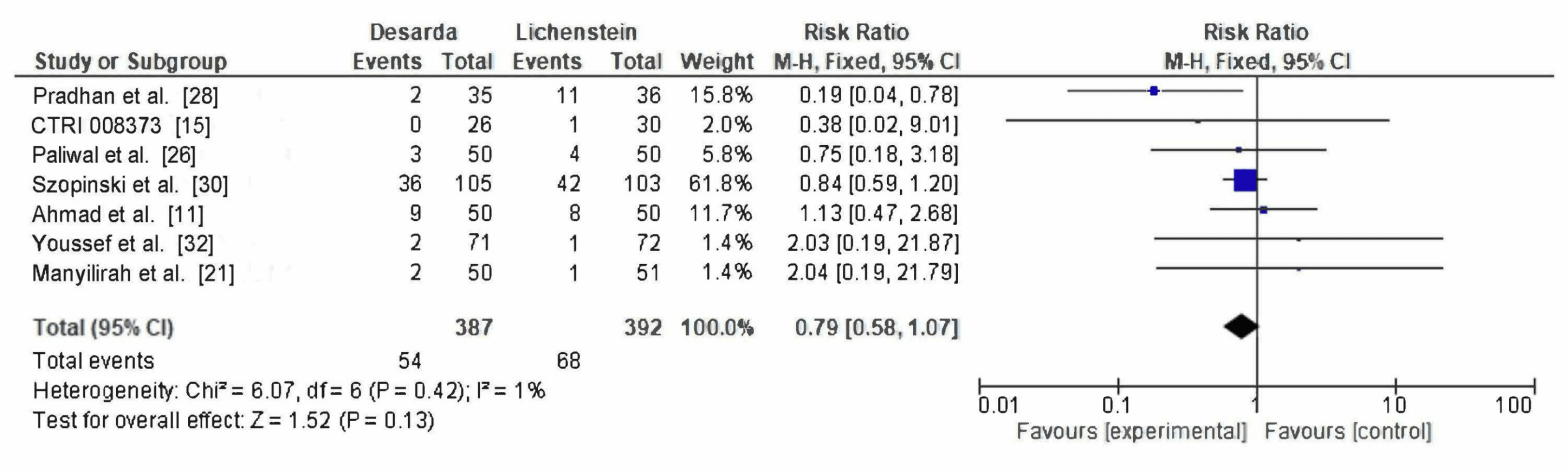
Comparison of loss of sensation reported between the Desarda technique and Lichtenstein mesh repair groups CTRI: International Clinical Trials Registry

Loss of sensation: Loss of sensation was evaluated in seven RCTs, reported in 54 of 387 patients in the Desarda group and 68 of 392 patients in the Lichtenstein mesh repair group. The RR was 0.79 (95% CI: 0.58-1.07; p = 0.13), with low heterogeneity (I² = 1%), as shown in Figure 11. Although the data are statistically reliable, the findings are not conclusive. A slight trend in favor of the Desarda technique is observed, but it does not reach statistical significance.

All postoperative outcomes are summarized in Table 2, which presents the certainty of evidence according to the Grading of Recommendations, Assessment, Development and Evaluation (GRADE) approach. 

**Table 2:** Summary of postoperative complications of the Desarda technique and Lichtenstein mesh repair groups RCT: randomized controlled trial; GRADE: Grading of Recommendations, Assessment, Development and Evaluation

Outcome	No. of RCTs	Patients (Desarda technique vs. Lichtenstein mesh repair)	Pooled risk ratio (95% CI)	Certainty of evidence (GRADE)
Chronic postoperative pain	7	357 vs. 354	0.26 (0.15–0.45)	Moderate (downgraded for study limitations)
Foreign body sensation	8	433 vs. 437	0.61 (0.42–0.88)	Moderate (downgraded for imprecision)
Seroma	15	772 vs. 776	0.68 (0.47–0.99)	Moderate (downgraded for study limitations)
Scrotal edema	11	549 vs. 549	0.52 (0.34–0.78)	Moderate (downgraded for imprecision)
Recurrence	9	523 vs. 525	0.76 (0.42–1.37)	Low (downgraded for imprecision & short follow-up)
Wound infection	17	811 vs. 842	0.61 (0.37–1.02)	Low (downgraded for imprecision & inconsistency)
Wound hematoma	13	708 vs. 735	0.96 (0.62–1.47)	Low (downgraded for imprecision)
Loss of sensation	7	387 vs. 392	0.79 (0.58–1.07)	Low (downgraded for imprecision & reporting variability)

Discussion

Tissue-based techniques such as the Bassini and Shouldice repairs have been utilised for many decades in the surgical management of inguinal hernia and remain important milestones in the evolution of hernia surgery. While both techniques have demonstrated acceptable recurrence rates in experienced hands, they are inherently associated with certain limitations. These include the creation of tension along the suture line, which can predispose to postoperative discomfort and recurrence, as well as the requirement for meticulous and often extensive dissection of an already weakened posterior inguinal wall [1,10-11]. The advent of prosthetic mesh repair, exemplified by the Lichtenstein mesh repair technique, sought to overcome these shortcomings by enabling a tension-free reconstruction of the inguinal canal. However, mesh implantation introduced a new spectrum of complications, including mesh infection, seroma formation, scrotal edema, foreign body sensation, chronic postoperative pain, and, in rare cases, mesh migration or erosion into adjacent structures.

The Desarda technique represents a mesh-free alternative that seeks to provide the advantages of tension-free repair while potentially reducing complications associated with synthetic materials. This method utilizes a 2 cm wide strip of the external oblique aponeurosis, which is sutured to reinforce and restore the posterior wall of the inguinal canal [1]. By using autologous tissue, the technique eliminates the foreign body reaction and mesh-related sequelae while maintaining structural integrity and physiological dynamics of the inguinal canal. Furthermore, it is relatively simple to learn, requires minimal additional dissection, and may be particularly advantageous in resource-limited settings where mesh availability or affordability is an issue.

This systematic review compared postoperative complications associated with the Desarda and Lichtenstein mesh repair techniques for primary inguinal hernia. Our findings indicate that the Desarda technique is associated with lower rates of chronic postoperative pain, foreign body sensation, seroma formation, and scrotal edema when compared with the mesh-based Lichtenstein repair. These advantages may be attributed to the avoidance of synthetic mesh implantation in the Desarda repair, which eliminates mesh-related inflammatory reactions and potential nerve irritation, both of which have been implicated in the development of chronic postoperative groin pain and foreign body sensation. While there was also a trend favoring the Desarda technique in terms of reduced recurrence, wound infection, wound hematoma, and sensory loss, these differences did not reach statistical significance, suggesting that both procedures remain comparable in these specific outcomes in the short to medium term. These results are in agreement with the general trend reported in several previously published studies, which have similarly demonstrated reduced postoperative discomfort and selected complication rates with Desarda repair, without compromising recurrence outcomes [32-35].

The GRADE assessment indicated moderate certainty for outcomes such as chronic postoperative pain, foreign body sensation, seroma, and scrotal edema, while evidence for recurrence, infection, wound hematoma, and sensory loss was of low certainty due to methodological limitations and short follow-up. Although Desarda may reduce certain mesh-related complications, further high-quality RCTs with longer follow-up and integrated cost-effectiveness analyses are needed, particularly to guide practice in resource-limited settings.

A comprehensive and methodologically rigorous search strategy was employed to identify all relevant studies published since the introduction of the Desarda technique in 2001. Multiple major databases were systematically searched, and strict inclusion criteria were applied to ensure that only high-quality evidence was incorporated. To minimize selection bias, performance bias, and confounding, only RCTs were included, while non-randomized comparative studies and previously published systematic reviews were excluded. This approach prevented duplication of primary data, reduced statistical heterogeneity, and enhanced the internal validity and reliability of the findings. The included trials demonstrated a low risk of bias, which further strengthened the credibility and reproducibility of the pooled estimates. By synthesizing the best available evidence, this review provides a robust comparison between the Desarda and Lichtenstein mesh repair techniques, offering clinically meaningful insights into postoperative outcomes, recurrence rates, and complication profiles. Consequently, this work contributes significantly to the existing body of literature on inguinal hernia repair and may inform both surgical decision-making and future research directions in this field.

Limitations

This systematic review has several limitations. Firstly, the majority of included RCTs featured relatively short follow-up periods, thereby limiting the ability to draw definitive conclusions regarding long-term recurrence rates and late postoperative complications. Secondly, there was notable heterogeneity in methodological quality across studies, with several trials lacking detailed reporting on randomization procedures and blinding, introducing potential risks of selection and performance bias. Furthermore, the evidence base demonstrated geographic and temporal limitations, as most trials were conducted in South Asia within the past decade, which may affect the generalizability of findings to other healthcare settings. Sensitivity analyses were considered to assess the robustness of the results; however, the limited number of studies available for certain outcomes precluded meaningful subgroup or exclusion analyses. Finally, publication bias was not formally assessed due to the small number of trials (<10) reporting on individual outcomes, rendering funnel plot analysis and related statistical tests underpowered and potentially misleading.

## Conclusions

This systematic review suggests that the Desarda technique offers a comparable and, in some aspects, favorable postoperative outcome profile when compared to the Lichtenstein mesh repair for primary inguinal hernia. Specifically, it is associated with a lower incidence of chronic postoperative pain, seroma, scrotal edema, and foreign body sensation. Although other complications were reported, the differences were not statistically significant. Given the comparable efficacy and reduced mesh-related morbidity, the Desarda technique presents a viable alternative, particularly in resource-limited settings. However, further high-quality RCTs with larger sample sizes are necessary to confirm these findings and inform surgical decision-making.
